# Adoption of electronic cards using Wi-Fi platform services by clients of banking sector during COVID-19 pandemic

**DOI:** 10.1177/18479790221112797

**Published:** 2022-07-11

**Authors:** Ali Matar, Abdelbaset M Alkhawaldeh

**Affiliations:** 1 School of Business, Dean of Scientific Research, 144850 Jadara university, Irbid, Jordan; 2 College of Business Administration, 123305 Taibah University, Medina, Saudi Arabia

**Keywords:** Usefulness, ease of use, awareness, bank credibility, reference group, security concerns

## Abstract

Wi-Fi technology is one of the most electronic services that recently emerged in the banking sector that provides significant benefits to clients and banks. Thus, the study aims to validate the factors influencing the adoption of Wi-Fi (Near Field Communication) technology by banking sector clients using an extended technology acceptance model. One hundred sixty-two valid surveys were gained from Jordanian clients via an online survey. Data were analyzed using the PLS-SEM technique. Perceived usefulness, perceived ease of use, and perceived bank credibility all have a significant effect on the adoption of Wi-Fi technology services. However, perceived awareness, reference groups, and security concerns have no significant effect on the adoption of Wi-Fi technology services. The research has several implications for theory and practice. It also makes several suggestions for future studies and points out some limitations.

## Highlights

1. The study examined the factors influencing the adoption of Wi-Fi technology services by banking sector clients using an extended technology acceptance model.
2. The study employed the PLS-SEM technique^1^ to analyze the results.
3. The results suggested that perceived awareness, reference groups, and security concerns have no significant effect on the adoption of Wi-Fi technology services.
4. The research has several implications for theory and practice. It also makes several suggestions for future studies and points out some limitations.

## Introduction

With the technological development of societies, especially in recent decades, electronic payment methods have become a requirement of the times due to the ease of payment, saving time and effort for clients. The need for electronic payment methods and financial technology has become urgent, especially recently with the emergence of the COVID-19 pandemic. The emergence of new technological means in any society must be accompanied by some challenges to implementing these modern means, as they are not used by all customers. Many factors must be taken into account thaccompaniesany the acceptance of financial technology and electronic payment, such as customer confidence and trust and the degree of safety in using these technologies. Among these recent divorces, this study will focus on the most common technologies that power contactless payments are Near Field Communication (NFC) and electronic payment using Wi-Fi service without the need for Pincodecode by the customer and their consequent risks.

The COVID-19 outbreak has hastened the adoption of mobile contactless payments, which are already a popular digital banking service. Retailers are working on ways to reduce physical contact cash usage. Customers can pay with their debit/credit cards or smartphone by just tapping it against a POS terminal (or other contactless readers). For both merchants and customers, it provides a quick, simple, and secure payment option. QR codes and NFC are the most common contactless payment systems. To allow contactless payments, mobile devices offer several security features, such as a unique code that verifies the device’s authenticity, the usage of tokens rather than card data, and biometric technologies. Another e-banking service is Bulk payment which is mostly utilized by corporate SMEs of banks, allowing them to send money to several recipients from a single account at the same time. These payments are ideal for corporate transactions like wage payments, paying contractors, suppliers, and dividends, as well as transactions of the government.^2^

In today’s banking climate, P2P (Peer-to-Peer) is prospering. It’s a straightforward payment system that lets people send money instantaneously from any device. Users can send money to another part by their computer or phone using a connected bank account, a credit/debit card, or a wallet stored-value account with only a few taps. P2P transactions should be supported by a high level of security and fraud measures, such as biometrics, PINs, or one-time passwords (OTPs) (One Time Password).

With the availability of digital devices (etc. P2P money transfers; QR codes; and NFC), banks around the world have offered an alternative method of business for their clients. This type of service is called e-banking services, which integrate the characteristics of conventional banking, social computing, and the Internet into one simple service.^3–5^ It should be noted that electronic services allow banks to communicate with customers and stakeholders effectively. Such services are also relatively inexpensive and are typical of high quality.^6–10^ Following these benefits, e-banking services have become one of the most important methods of service in the financial industry. Nevertheless, some of the banks’ customers are still hesitant to use this type of service.

Without understanding the deeper reasons behind these reluctances on online banking services, the Jordanian banks will continue to have issues with the implementation of e-banking services, because their customers are less interested in and motivated by this new banking technology.^11,12^ The factors behind that may are mostly related to the individual’s acceptance of the technology. Therefore, research based on the Technology Acceptance Model (TAM) might shed some light on this matter. It should be noted that the TAM has been extended over the years.^13^ Nevertheless, research based on the TAM are mostly conducted in developed countries, invalidating its generality in other countries.^12,14,15^

As a result, it may be claimed that the largest hurdle to this technology’s success is persuading people to utilize it as a full replacement for existing traditional channels.^16^ In fact, as e-banking is in the early stage of its implementation in Jordan, quite a few numbers of researchers (i.e., Refs. 11, 12, 15, 17–19) have addressed the related issues of such technology. Even though these studies enriched the understanding the main predictors of the adoption of e-banking in Jordan, there are other relevant factors such as payment security and the Bank Credibility, Group Influence on the intention to use e-banking services more explanation is required in the Jordanian context. As a result, the e-banking literature gap may be summarized as a need to propose a parsimony conceptual model that can accurately define e-banking adoption from the standpoint of Jordanian clients. Hence, to fill this gap, this study aims to assess the applicability of the AM extended model in developing countries, such as Jordan, by evaluating the impact of perceived usefulness, perceived ease of use, perceived awareness, perceived bank credibility, and reference group influence on the intention to use e-banking services. Based on the results, an extended model was proposed. The current research demonstrated that the proposed extended model is applicable to banking industries in developing countries. This is a useful discovery as it can assist the banks, clients, and policymakers in drafting strategies that can attain and sustain their growth.

The payment’s several innovative payment solutions goal is to enable the clients to pay securely and seamlessly. Our motivation to write this study stems from the problem that we faced as well as many clients face when they used their Debit or Credit Cards. Many banks have recently introduced an electronic payment service via Wi-Fi (NFC) technology without any need to enter a password or pin code. Nevertheless, customers can pay directly by simply passing the card in different places such as retail stores and shopping malls to pay for their purchases. By using this technology, clients can save time and effort and there is no need to touch the payment machine and thus reducing the spread of infectious diseases such as COVID-19. However, the problem lies in the high risks resulting from that when losing this card, any person holding this card can withdraw immediately as soon as it is passed to the merchant and therefore he can easily carry out payments without any password. This poses a risk to the client’s account if his card is lost. Although, the banks set a ceiling or limit for daily withdrawals using this service of 50 dinars, this constituted a very dangerous use of this technology, which is Wi-Fi (NFC) technology, and it prompted many customers to stop this service, including us.

Another constraint that may reduce the percentage of using Credit/Debit cards whether with Wi-Fi (NFC) technology services or not, the commission that is conducted (0.5JD) from the client account for every single payment using card purchase. In addition to the previous constraint, habits (use of cash), limited trust for financial institutions, and financial literacy lack of awareness are major factors that may impact the use of Credit/Debit cards in Jordan.

Consequently, this problem is in line with previous literature problems stating that the large amounts of money being invested in banks have not resulted in adequate level of the adoption of e-banking services in Jordan as it is not in line with what was expected to benefit from these financial technologies.^11,12,17,20^

What enhances the existence of this problem, is that prior to 2017, there were no general laws relating to client protection in Jordan and no specific financial customer protection regulations apart from the 2012 Instructions on Dealing with Customers Fairly and transparently that only covered customer’s bank accounts. In 2017, the central bank of Jordan developed the regulations by introducing “Instructions on the Protection of Personal Data of the Clients of Payment Services and Electronic Transfer of Funds” for non-banks to safeguard customers’ money. These regulations help in developing data collection and customer complaints, customer protections rules, ensure systems governance and safety. Moreover, under the new licensing requirements, banks are required to submit a Policy for dealing with Customer Complaints in-line with the central bank instructions regarding customer complaints.

The study aims to verify the factors affecting the dominate of electronic financial services through the use of a developed model for this purpose and to propose solutions and recommendations to solve the problems of dealing with this modern financial technology. This study is considered one of the recent studies, despite there being many studies that dealt with the issue of electronic services and financial technology, but this study is considered the first study to address and focus on the Wi-Fi (NFC) technology service and its challenges in Jordan.

This paper is structured as follows: the next section presents the literature review, the overview of Jordanian e-banking services is included in section 3. section 4 explored the theoretical framework. Research methodology and data analysis are presented in sections 5 and 6 respectively. Section 7 states the conclusion and discussion. and eventually, the last section includes limitations and recommendations.

## Literature review

Despite there are a significant number of researches being done on the adoption of e-banking services by banks, and the factors influencing the clients’ use of the services. But we did not find any study focus on Wi-fi (NFC) technology services and its challenges. Most of the researches focus on either personal reasons, such as individuals’ perceptions of e-banking, or demographical influence. This section will review some studies relating to the factors influencing the use of e-banking services by the customers. Individuals’ perceptions of Internet banking may influence their intentions in using the services.

The word ‘trust’ has been mentioned a few times across the literature as abovementioned, while other literatures have also mentioned this reason as well. Management students mentioned that trust propensity, among other reasons mentioned before, affects their inclinations in using the e-banking services.^21^ This study was supported by Ref. 22 study of Iranian firms, which hailed trust as most pertinent in implementing Internet banking. Further support was provided by Ref. 23 in their study in Mauritius. Meanwhile, the Internet-only banks (IOBs) in France cultivated their consumers’ trust through quality websites, respectful reputation, relative advantage, structural assurance, and consumers’ familiarity with Internet banking.^24^ Moreover, the academics in Ref. 25 study admitted that the banks’ e-banking’s performance expectancy, effort expectancy, and perceived risk affect their motivation in using Internet banking. From this, it can be observed that the external factor may also influence the use of Internet banking services.

In Jordan, Ref. 15 found that perceived bank credibility, perceived ease of use, reference group influence, perceived awareness, and perceived usefulness had a positive impact on the use of mobile banking services by the customers. Baabdullah et al.,^26^ tested the impact of the predictors of client intention of self-service technologies and its adoption. They suggested significant impact of the tested predictors in various ways due to the moderating impact of channel type. Moreover, Ref. 27 analysed factors impacting Jordanian customer’s adoption of e-banking services. They proposed that perceived risk, effort expectancy, hedonic motivation is significantly impacting the customer intention, while social influence does not have a significant impact on customer behavioural intention. AlKailani, M.^28^ demonstrated that the integration of three new constructs – Perceived Risk (PR), Perceived Trust (PT), and Bank Credibility (BC) – will increase the model’s reliability in predicting the use of e-banking services. This was supported by Ref. 29. Furthermore, it was observed that web privacy,^30^ Internet experience and enjoyment,^31^ and account essential factor^32^ affect the adoption of Internet banking by the customers. It is also worth noting that demographic factors also influence the adoption of e-banking services, as demonstrated by Ref. 33. Ahmad et al.,^34^ also examined factors that expected to improve the intention of customers to use Arabic e-commerce websites.

Ghali, Z.^6^ investigated the client’s loyalty toward e-banking services as well as the e-trust. He found significant impact of website design and responsiveness on e-trust and satisfaction. Ramesh et al.,^7^ suggested that responsiveness, ease-of-use, reliability, dependence, comfort, secured transaction, and efficiency had significant impact on e-loyalty and customer satisfaction.

Putit et al.,^35^ suggested that fear, perceived usefulness on consumers’ attitude, and convenience have significant impact on consumer attitude towards contactless payment usage. On the other hand, social influence, trust, perceived ease of use have insignificant impact.

Abdul Rais et al.,^36^ found that the most favorable method of payment for the case of university students is cashless payment and e-wallet definitely. However, the study found that student used OTP to secure their digital payments, and they revealed high level of awareness regarding the using of public Wi-Fi, security of software, and threats.

Dubey et al.,^37^ proposed that artificial intelligence and innovative thinking have fueled contactless banking payments, besides, how these developments promote social distancing in COVID-19 period. They argued that people can transact business as usual and get services in the comfort of their homes by using these technologies and it will be indeed the future of the world economy, especially during the lock down pandemic times with no interactions among people.

Karjaluoto et al.,^38^ suggested that the UTAUT2 model explain 70% of the variance in usage intention of contactless payment systems in developed country like Finland. They found that consumers’ overall satisfaction and habit have significant impact on usage intentions besides the positive relationship between use and intention.

Chaimaa et al.,^3^ reviewed service highlighting various aspects regarding e-banking services challenges and risks, and discussing some proposed solutions. They proposed that security concerns, one of the biggest issues that faced the use of e-banking is security of transactions. Banking customers’ worry that their accounts might get hacked or accessed by unauthorized people. There is also a fear that the funds they transfer may not reach the intended recipients. Chauhan^4^ found that perceived risk, security information availability, and consumer innovativeness had significant impact on customer intention and adoption of using e-banking services in India.

New factors of navigation and interaction have been mentioned by Ref. 39, they found that customers give high priority to these usability factors of navigation and interaction rather than functionality. Hu et al.,^40^ introduced the Fintech services by providing more comprehensive sight of the determinants of user’s, they found that user’s trust in Fintech services has a very significant influence on user’s attitudes for adoption. Next to the TAM model Ref. 41 proposed task-technology fit (TTF) model, their results supported the impact of perceived privacy, perceived security, perceived usefulness and TTF on the customers’ continued intention to use mobile banking. Keramati et al.,^42^ provided an appropriate framework to compensate e-bank errors on an Iranian private bank by investigating the relationship between customer satisfaction, service recovery, and service failure.

In their study on the bank customers of the urban area of coastal Karnataka, Ref. 43 observed that customers adapted Internet banking if they were provided with positive perception and driving factors. Apart from that, they also stated that customers’ who were equipped with knowledge, resource, security and privacy were more open to new technology. Their study was supported by research by Ref. 44, who adopted a TAM-diffusion theory model (TAM-DTM) in studying the Malaysian banking sector. From their study, trust, cost, security, and privacy can impact customers’ motivation in using the online services, and these constructs can be integrated within the TAM-TDM.

In a similar vein, Ref. 45 who examined this issue in Gujarat using the TAM, reiterated the significant role of customers’ perceptions in promoting the use of e-banking. Their study demonstrated that individuals’ impressions on Internet banking’s security, usefulness, ease of use, and social influence directly impacted the use of this service. Roy et al.,^46^ drew the same conclusion from their study that was based on TAM and perceived risk theory in India; perceived ease of use and external risk influence the use of Internet banking by the customers. Other than the factors mentioned in this paragraph, Nasri & Charfeddine,^47^ stated that the Tunisians’ government support, technology support, self-efficacy, perceived behaviour control, and personal attitudes determined their use of Internet banking services.

Other researchers have stated similar reasons. For example, a study in Hong Kong of retail customers’ Internet banking behaviour revealed that personal innovativeness and perceived risk had an impact on the adoption of e-banking.^48^ Meanwhile, in terms of self-efficacy, Wang et al. demonstrated that personal abilities may have a role in influencing ones’ impression on mobile banking’s credibility, usefulness, and ease of use. A study by Ref. 49 provided an interesting insight on the links among age, technology acceptance (TA), and mobile banking adoption in Indian consumers. This study demonstrated the significant differing attitudes toward mobile banking across three segments – the TA leaders had the most positive attitudes towards Internet banking, followed by TA followers and TA laggards, in that order. Interestingly, age influence TA and its usage.

While internal factors may affect the adoption of Internet banking, external factors may also influence its usage. Karthikeyan, P.^50^ observed the situation in the banks in Coimbatore city. From this study, the employees’ work satisfaction was found to affect the delivery of service; unsatisfied employees provided sub-par services, discouraging consumers’ from opting for any type of services. In a similar vein, the e-Government Adoption Model (GAM) demonstrated that the motivations for using Internet banking differ according to the phases of services (interaction, static and transaction).^51^ Furthermore, Ref. 52 indicated that large and young firms with a diverse management board were more likely to adopt Internet banking services, especially those who were competing with a large number of firm users. These are some of the external factors influencing the adoption of e-banking.

Based on this literature review, there seems to be a lack of knowledge on the adoption of e-banking services in developing countries, especially that focused on security concerns. Following this, an extended model of the TAM was constructed to strengthen the model, as proposed by previous scholars.^4,21,28,44,45,51,53–55^ This study utilised two constructs of the TAM and integrated four new constructs into the model, which were security concerns, perceived awareness, perceived bank credibility, and reference group influence. The new constructs were based on suggestions from previous literature.^3,21,28,51^ This framework will test its applicability in developing countries such as Jordan, enriching the body of knowledge on e-banking services.

## An overview of Jordanian E-banking services

Despite the Jordanian commercial banks had launched e-banking services in 2012,^56^ the percentage usage of these services among their costumers still very low, where only 8% of the customers have performed their banking operations via e-banking services.^57,58^ So, Jordanian banks are facing some difficulties in implementing their e-banking services because most of their clients prefer the conventional banking method. Some studies have looked into this matter, and they observed that most the bank customers’ in Arab countries took quite some time before starting to use the Internet and online banking applications.^59,60^ The Arabs were also observed to have social, cultural, and economic reservations about e-banking services.^47,61–63^

**Figure 1.**
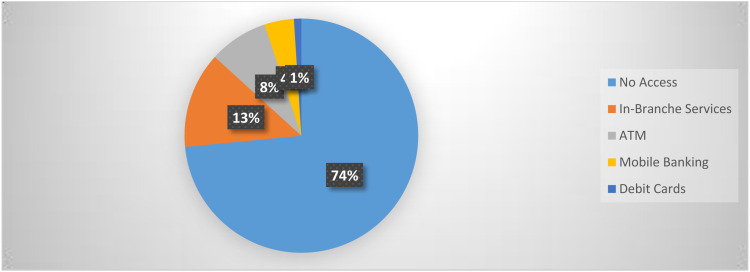
Financial inclusion & payments in Jordan. Source: (USAID, Digital finance country report Jordan, 2019).

According to Jordan’s financial inclusion, about 58% of the Jordanian population is unbanked. Figure 1 reveals that payments in Jordan divided into credit/debit cards 1% only which is very low, 8% ATMs, mobile banking 4%. In addition, 13% use in-branch services, and 72% of micro-businesses don’t access any financial services. Usage of digital financial services by micro-businesses was very low, 6% for Dinarak, 4% for Mahfazti, 7% for eFAWATEERcom, 6% for Zain Cash, and 2% for Aya-Pay. Service used include bill payment, check balances, and receive/send transfers and, pre-pay airtime/subscriptions. The overwhelming majority of micro-businesses preferred dealing with cash which is faster and easier.^64^

Low financial and technological literacy plays a key role in the limited use. Enhanced ease of use, functionality and promotion of internet and mobile banking platforms could contribute to higher usage rates. The rate of credit card ownership (4.8%) is much lower than debit card ownership, but credit cards are much more used than debit cards. 5.5% of adults in Jordan used the internet to buy something online or pay bills online in 2017, up from 2.5% in 2014. Increased ownership and use of card products could lead to higher rates of online payments, as could increase usage of internet banking and mobile banking services offered by banks. Mobile banking is more popular than internet banking, although neither is used widely yet. Just 1.4% of adults had internet banking and 2.1% had mobile banking, and usage of these services (at least once per year) was even lower at 1.2% and 1.6% of adults, respectively.^65^

According to central bank of Jordan, there are some regulatory constraints impacting growth of digital financial services, such as: First, 5% income tax for providers levied on Fin-tech companies versus 35% for financial services. Second, high rate of the payment service providers (PSP) capital of 2 million JD compared with 370,000 $ in the European countries. Third, the mobile wallet transactions are still low (limits: P2P-500JD, withdrawal1,000JD) it is lower than African and European countries. Fourth, the prohibition of Near Field Communication (NFC) by central bank of Jordan prevents the development of e-banking such as mobile banking. Fifth, the lack of E-signature.

## Theoretical framework

**Figure 2.**
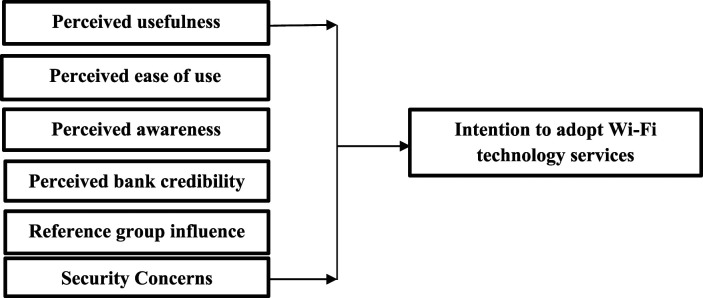
Proposed model.

The model (Figure 2 below) used in this work was designed, based on past literature to assess the following hypotheses.

H1 Perceived usefulness positively impacts the adoption of use Wi-Fi Services.

H2 Perceived ease of use positively impacts the adoption of use Wi-Fi Services.

H3 Perceived awareness positively impacts the adoption of use Wi-Fi Services.

H4 Perceived bank credibility positively impacts the adoption of use Wi-Fi Services.

H5 Reference group influence positively impacts the adoption of use Wi-Fi Services.

H6 Security concerns influence positively impacts the adoption of use Wi-Fi Services

## Research methodology

The study used an online survey with evidence from 162 bank clients in Jordan to assess their opinions on the adoption of Wi-Fi technology services. Three questions of perceived usefulness; two questions of perceived ease of use; three questions of reference group influence and two questions of intention to adopt Wi-Fi technology services,^21^ three questions of perceived awareness,^51^ four questions of perceived bank credibility,^28^ and six questions of security concerns.^66^ (Table A1 scale items). The statistical analysis was carried out using structural equation modeling (Smart PLS 3.0).

## Data analysis

The socio-demographic information of the participants is shown in Table 1. Moreover, smart PLS 3.0 was used for data analysis. To attain convergent validity, Hair et al.,^67^ stated that the factor loading should be 0.70 and above, whereas other scholars claimed that loadings must be more than 0.5.^68^ Moreover, the average variance extracted (AVE) “is the grand mean value of the squared loadings of a set of indicators”^68^ should be 0.50 or higher. Furthermore, Composite reliability (CR) and Cronbach’s Alpha 0.70 and above. Fornell and Larcker’s^69^ criterion was also used to assess discriminant validity: “this method states that the construct shares more variance with its indicators than with any other construct, to test this requirement, the AVE of each construct should be higher than the highest squared correlation with any other construct” (p.112). Convergent and discriminate validity was achieved because they exceeded the presented criteria, as shown in Tables 2 and 3.^70–72^ Besides that, the results showed that the level of R Square (R^2^) (*R*^*2*^ = 0.571) met^73^ requirements. Table 4 demonstrates the bootstrapping and path coefficient outcomes of the correlations.

**Table 1.** The profile of the participants.

Variables	Descriptions	Frequencies	Percentages, %
Gender	Male	127	78.4
	Female	35	21.6
Age	18–25	43	26.5
	26–45	69	42.6
	46–60	34	21
	61 and more	16	9.9
Academic qualification	High school and less	13	8
	College degree	6	3.7
	Bachelor’s degree	89	55
	Master’s degree and more	54	33.3
Do you use bank cards for shopping?	Yes	133	82.1
	No	29	17.9
If yes, how many times per month do you use it?	1–5	86	53.1
	6–10	31	19.1
	11–15	15	9.3
	16 and more	30	18.5
Does your card support the wi-fi feature in the payment?	Yes	106	65.4
	No	56	34.6
If yes, do you use this feature to pay?	Yes	93	57.4
	No	69	42.6

**Table 2.** Convergent validity.

Variables	Item	Loading	Average variance extracted	C. Alpha	Composite reliability
Perceived usefulness	PU 1	0.891	0.726	0.812	0.887
	PU 2	0.809			
	PU 3	0.855			
Perceived ease of use	PEU 1	0.859	0.798	0.753	0.888
	PEU 2	0.927			
Perceived awareness	PA 1	0.873	0.712	0.796	0.881
	PA 2	0.777			
	PA 3	0.878			
Perceived bank credibility	PBC1	0.850	0.677	0.842	0.894
	PBC2	0.814			
	PBC3	0.790			
	PBC4	0.837			
Reference group influence	RGI1	0.814	0.671	0.758	0.859
	RGI2	0.761			
	RG13	0.879			
Security concerns	SC1	0.700	0.510	0.807	0.861
	SC2	0.647			
	SC3	0.757			
	SC4	0.816			
	SC5	0.666			
	SC6	0.686			
Intention to adopt wi-fi technology services	IA1	0.905	0.826	0.789	0.905
	IA2	0.912			
23 items

**Table 3.** Discriminant validity.

	PA	PBC	PEU	IA	RGI	SC	PU
PA	0.844						
PBC	0.501	0.823					
PEU	0.648	0.582	0.893				
IA	0.576	0.617	0.658	0.909			
RGI	0.574	0.527	0.487	0.512	0.819		
SC	0.653	0.705	0.700	0.616	0.665	0.714	
PU	0.509	0.591	0.509	0.598	0.509	0.638	0.852

**Table 4.** Path coefficient of hypotheses.

H	Relationship	Std. Beta	Stander error	t-Value	Results	*p*-value
H1	PU -> IA	0.231	0.107	2.165	Accepted	.016
H2	PEU -> IA	0.334	0.114	2.936	Accepted	.002
H3	PA -> IA	0.123	0.097	1.270	Rejected	.104
H4	PBC -> IA	0.229	0.133	1.724	Accepted	.044
H5	RGI -> IA	0.087	0.096	0.905	Rejected	.184
H6	SC -> IA	−0.070	0.133	0.527	Rejected	.300

## Discussion and implications

The current study sought to identify the factors that influence the adoption of Wi-Fi services in Jordan. Per the empirical findings, three factors influence clients' intent to use Wi-Fi services. **H1**, **H2**, and **H4** described the relationships between these factors and the adoption of Wi-Fi services. Each of these assumptions was supported, implying that Jordanian banking clients consider these variables crucial when deciding whether or not to use Wi-Fi services.

Perceived usefulness, perceived ease of use, and perceived bank credibility has a positive and significant association with the use of Wi-Fi (NFC) technology services. These findings are consistent with previous revisions e.g., Refs. 15, 41, and 45. These results confirmed TAM extended model and a planned behavior theory, which states that perceptions lead to behavior (use Wi-Fi services). Clients put a high priority on perceived usefulness, perceived ease of use and perceived bank credibility on their Wi-Fi technology services when conducting bank transactions.

This study found no significant relationship between perceived awareness and reference group influence with the use of Wi-Fi services, opposite to our assumptions. With this sample, the above findings are consistent with past studies e.g., Refs. 74–76. However, in conversely to others.^15,77,78^ In the Arab context, a lack of citizen awareness hampered the adoption of e-services, necessitating increased citizen awareness.^74^ According to the findings of this study, the participants have a poor understanding of the use of Wi-Fi (NFC) technology services because they lack knowledge about technology services. Banking sectors maybe do not raise customer awareness of their services. As a result, the study recommends that banks improve awareness among clients by utilizing the media. Moreover, one reason for reference group influence’s insignificance could be that clients prefer to make their own economic choices rather than consulting others.^76^

Hypothesis six refused, implying that the security concerns factor is insignificant and has a negative impact on Wi-Fi service use (= −0.070, t = 0.527). The findings contradict prior research by Ref. 76, who noticed that security is a big major worry when undertaking banking transactions via online channels. They revealed that security has a significant impact on mobile banking adoption. Clients demand banks enhance their security features, particularly when it comes to wireless networks, where they expect privacy protection and transaction security. This suggests that there may be additional factors involved in Wi-Fi (NFC) technology service use.

This research backs up and expands on the TAM model and planned behavior theory in the banking business, particularly in the Arab world. Based on the literature review, there appears to be a knowledge gap regarding the adoption of e-banking services in developing countries, particularly in terms of security issues. Based on that, an extended TAM model was built, as suggested by earlier experts, and four new components were included in the model. The novel constructs were developed based on earlier research.^3,21,28,51^

Moreover, although a large number of research on acceptance of e-banking services and the factors influencing clients are being undertaken. However, we were unable to find any studies that focused on the issues with Wi-Fi (NFC) technology services. The bulk of studies focused on either personal or demographic characteristics, such as people’s perceptions about e-banking.

Practically, the findings will assist practitioners in clarifying the status of Wi-Fi technical services in Jordan, as well as in developing strategies to speed up the use of Wi-Fi technology services in the banking industry. In particular, compared to other developed countries, Jordan’s adoption of Wi-Fi technical services is still in its early stages.

## Limitations and recommendations

This study had limits in terms of sampling, sample size, and variable type. An online survey has inherent limitations.^79^ Since some people do not want to participate in any activities during COVID-19, particularly the online links, we also posted the link face-to-face at banks to get replies from people. Furthermore, researchers did not assess how well each participant comprehended each question; therefore, it is conceivable that some participants did not fully get the items' meaning. Thus, a more representative sampling method will be required to investigate the clients’ perspectives on the adoption of Wi-Fi (NFC) technology in the future. Future research should use a larger sample size and the random sampling method to test the proposed research framework. Furthermore, future studies should test other potential variables to broaden this model by including moderating or mediating variables in different contexts. The study recommends to the PSP and banks to adopt new technology to solve the constraint of security concerns for clients such as using what called dash-cam^80^ capture face images and voice commands then the merchandise payment machine can recognize the clients face or voice. Moreover, services providers can integrate digital assistants, such as Siri and Alexa, in infotainment systems that can be used for making voice for payments. The study also recommends for the banks in Jordan to stop deducting the commission for e-banking payment whether via credit/debit cards or mobile-services to encourage clients to use these kinds of services. Its recommended to central bank of Jordan to exist new regulations and legislation that protect the clients against any hacking or fraud risk relates to e-banking services.

In conclusion, the results of this study come on the opposite of authors assumptions and expectations since perceived usefulness, perceived ease of use, and perceived bank credibility has a positive and significant association with the use of Wi-Fi (NFC) technology services. Moreover, the study results revealed insignificant relationship between perceived awareness and security concerns and Wi-Fi service use. These results are optimistic for banks and policy makers reflect the ability and readiness of consumers in Jordan to use the fourth industrial revolution tools such as Fin-tech, contactless payment, E-wallets, and all kind of digital payments and applications.

## Supplemental Material

Supplemental Material - Adoption of electronic cards using Wi-Fi platform services by clients of banking sector during COVID-19 pandemic Click here for additional data file. Supplementary Material for Adoption of electronic cards using Wi-Fi platform services by clients of banking sector during COVID-19 pandemic by Ali Matar and Abdelbaset M Alkhawaldeh in International Journal of Engineering Business Management.
