# Supplementary material for: Adoption of electronic cards using Wi-Fi platform services by clients of banking sector during COVID-19 pandemic
Source: International Journal of Engineering Business Management. 2022 Jul 11;14:18479790221112797. doi: 10.1177/18479790221112797 (PMC9274143; doi:10.1177/18479790221112797)
Supplement: Supplemental Material - Adoption of electronic cards using Wi-Fi platform services by clients of banking sector during COVID-19 pandemic [file sj-pdf-1-enb-10.1177_18479790221112797.pdf]

**Appendix**

**Table A1 Scale items**

**First Part**

1. **Gender**: A. Male ☐ B. Female ☐

2. **Age**: A. 18 - 25 and less ☐ B. 26-45 and less ☐ C. 46- 60 and less ☐ D. 61 and more ☐

3. **Academic qualification**:

A. High School and less ☐ B. College Degree ☐

C. Bachelor's Degree ☐ D. Master's Degree and more ☐

**4. Do you use bank cards for shopping?**

A. Yes ☐ B. No ☐

**5. If yes, how many times per month do you use it?**

A. 1 TO 5 ☐ B. 6 TO 10 ☐ C. 11 TO 15 ☐ D. 16 AND More ☐

**6. Does your card support the Wi-Fi feature in the payment?**

A. Yes ☐ B. No ☐

**7. If yes, do you use this feature to pay?**

A. Yes ☐ B. No ☐

**Second Part**

**Perceived usefulness (Kumar et al., 2017)**

1- Electronic cards using Wi-Fi service would save my travelling expenses to the bank.
2- Electronic cards using Wi-Fi service would be useful as it would save my time.
3- Electronic cards using Wi-Fi service would be useful because of its convenience to use anywhere.

**Perceived ease of use (Kumar et al., 2017)**

1- Learning to use electronic cards using Wi-Fi service would be easy for me.
2- Electronic cards using Wi-Fi service would provide me with easy user interface.

**Perceived awareness (Shareef et al., 2018)**

1- I am aware of electronic cards using Wi-Fi service.
2- I know the benefits of using electronic cards using Wi-Fi service.
3- I have gone through educational/training programs about the overall features of electronic cards using Wi-Fi service.

**Perceived bank credibility (AlKailani, 2016)**

1- The bank I deal with always keeps its promises.
2- I have a close friendly relationship with my bank personnel
3- My bank seldom makes mistakes regarding my bank transactions
4- My bank always provides me with all the help I need. Definitely, I will recommend it to my friends.

**Reference group influence (Kumar et al., 2017)**

1- I would show my social group that I use electronic cards using Wi-Fi service.
2- I would use electronic cards using Wi-Fi service if my social group uses it.
3- I would discuss the features of electronic cards using Wi-Fi service with my social group.

**Security Concerns (Gurung & Raja, 2016)**

1- I would not feel secure sending sensitive information across the electronic cards using Wi-Fi service.

2- Electronic cards using Wi-Fi service is not a secure means through which to send sensitive information.
3- I would not feel totally safe providing sensitive information about myself over the electronic cards using Wi-Fi service.
4- I think my sensitive information sent to online companies by electronic cards using Wi-Fi will be accessed by unauthorized parties.
**5-** I hesitate to make purchase from the electronic cards using Wi-Fi service because of security issues if i lost my card any unauthorized person can find it and use it without any passcode.
**6-** I contact my bank to stop the services of Wi-Fi for security concern purposes.

**Intention to adoption of Wi-Fi technology services (Kumar et al., 2017)**

1- I intend to use electronic cards using Wi-Fi service.
2- I predict that I shall use electronic cards using Wi-Fi service.
